# Circularly permuted tRNA genes: their expression and implications for their physiological relevance and development

**DOI:** 10.3389/fgene.2014.00063

**Published:** 2014-04-01

**Authors:** Akiko Soma

**Affiliations:** Graduate School of Horticulture, Chiba UniversityMatsudo, Japan

**Keywords:** tRNA gene, circular gene permutation, BHB motif, tRNA-splicing endonuclease, intron

## Abstract

A number of genome analyses and searches using programs that focus on the RNA-specific bulge-helix-bulge (BHB) motif have uncovered a wide variety of disrupted tRNA genes. The results of these analyses have shown that genetic information encoding functional RNAs is described in the genome cryptically and is retrieved using various strategies. One such strategy is represented by circularly permuted tRNA genes, in which the sequences encoding the 5′-half and 3′-half of the specific tRNA are separated and inverted on the genome. Biochemical analyses have defined a processing pathway in which the termini of tRNA precursors (pre-tRNAs) are ligated to form a characteristic circular RNA intermediate, which is then cleaved at the acceptor-stem to generate the typical cloverleaf structure with functional termini. The sequences adjacent to the processing site located between the 3′-half and the 5′-half of pre-tRNAs potentially form a BHB motif, which is the dominant recognition site for the tRNA-intron splicing endonuclease, suggesting that circularization of pre-tRNAs depends on the splicing machinery. Some permuted tRNAs contain a BHB-mediated intron in their 5′- or 3′-half, meaning that removal of an intron, as well as swapping of the 5′- and 3′-halves, are required during maturation of their pre-tRNAs. To date, 34 permuted tRNA genes have been identified from six species of unicellular algae and one archaeon. Although their physiological significance and mechanism of development remain unclear, the splicing system of BHB motifs seems to have played a key role in the formation of permuted tRNA genes. In this review, current knowledge of circularly permuted tRNA genes is presented and some unanswered questions regarding these species are discussed.

## Introduction

The cloverleaf structure of a single polynucleotide tRNA molecule is universally conserved among organisms. However, tRNA genes are often divided into parts on the chromosome; in bacteria, archaea, eukarya, and organelles, several tRNA genes are interrupted by various types of introns, which are removed by RNA splicing after transcription (Thompson and Daniels, 1988; Kjems et al., 1989; Westaway and Abelson, 1995; Belfort and Weiner, 1997; Marck and Grosjean, 2002, 2003; Jühling et al., 2009; Abe et al., 2011). Introns in nuclear and archaeal tRNAs are generally cleaved by tRNA-splicing endonuclease (Reyes and Abelson, 1988; Abelson et al., 1998; Calvin and Li, 2008), while those in eubacteria and organelle tRNAs are encoded as self-splicing group I or II introns (Kuhsel et al., 1990; Xu et al., 1990a; Reinhold-Hurek and Shub, 1992; Biniszkiewicz et al., 1994; Jacquier, 1996; Bonen and Vogel, 2001). In addition to these well-known *cis*-spliced tRNA genes (intron-containing tRNAs), recently developed software has enabled the identification of additional distinct types of disrupted tRNA genes. Use of the Split-tRNA-Search (Randau et al., 2005a), SPLITS and SPLITSX (Sugahara et al., 2006, 2007) packages, in combination with the widely used tRNAscan-SE program (Lowe and Eddy, 1997), has led to the discovery of a variety of disrupted tRNA genes from the archaeal lineage, such as *trans*-spliced tRNAs (split tRNAs) that are joined at several positions in the cloverleaf structure (Randau et al., 2005a,b; Fujishima et al., 2009; Chan et al., 2011) and *cis*-spliced tRNAs containing one or multiple introns at non-canonical positions (Sugahara et al., 2008, 2009; Chan and Lowe, 2009). These newly identified tRNAs commonly harbor a characteristic bulge-helix-bulge (BHB) motif, which comprises two 3-nucleotide bulges separated by a single 4-base pair stem and was originally identified around the intron-exon junction of eukaryal and archaeal tRNAs (Thompson and Daniels, 1988; Kjems et al., 1989; Belfort and Weiner, 1997; Fabbri et al., 1998; Marck and Grosjean, 2003).

Nuclear tRNA introns are generally short, comprise a relaxed form of the BHB motif denoted as a hBH or BHB-like (BHL) motif, and are located exclusively between positions 37 and 38 (37/38), which is 3′ adjacent to the anticodon (the canonical position) (Marck and Grosjean, 2002, 2003; Jühling et al., 2009). This limited location of the BHB motif in the cloverleaf structure is crucial for the precise recognition of precursor tRNAs (pre-tRNAs) by eukaryal tRNA-splicing endonucleases (Greer et al., 1987; Reyes and Abelson, 1988; Westaway and Abelson, 1995; Di Nicola Negri et al., 1997; Trotta et al., 1997, 2006; Xue et al., 2006; Calvin and Li, 2008). However, recent analyses of the nuclear and nucleomorph genomes of unicellular algae identified a number of non-canonical BHB-mediated disrupted tRNA genes, including circularly permuted and atypical intron-containing genes (Kawach et al., 2005; Soma et al., 2007, 2013; Landweber, 2007; Maruyama et al., 2010; Chan et al., 2011). Analysis of the processing intermediates of permuted tRNAs revealed a new strategy for post-transcriptional processing of genetic information by inversion of RNA fragments and relocation of the termini via circularization of pre-RNA molecules (Soma et al., 2007, 2013; Maruyama et al., 2010). A further analysis also identified permuted tRNA genes in an archaeal lineage (Chan et al., 2011), highlighting the considerable diversity and wide distribution of tRNA gene disruption among organisms. While BHB motifs and the tRNA-intron splicing system must have been a prerequisite for the development of permuted tRNA genes (Soma et al., 2007; Sugahara et al., 2009; Maruyama et al., 2010; Tocchini-Valentini and Tocchini-Valentini, 2012; Kanai, 2013), their detailed mechanisms and physiological relevance remain unclear.

Here, the structure, expression, and phylogeny of circularly permuted tRNA genes are summarized. Discussions of their possible physiological relevance and method of development in correlation with the tRNA expression system and other disrupted non-coding RNA genes are also provided.

## Identification and distribution of circularly permuted tRNA genes

Circularly permuted tRNA genes were initially identified in the nuclear genome of *Cyanidioschyzon merolae* 10D (Soma et al., 2007), an ultra-small unicellular red alga that inhabits an extreme environment (pH 1–3, 40–50°C) and represents one of the most ancestral forms of eukaryote (Kuroiwa, 1998; Nozaki et al., 2003, 2007; Matsuzaki et al., 2004). A primary search of the complete 16.5 Mbp *C. merolae* nuclear genome sequence was performed using tRNAscan-SE, the most well-known and widely used software, which identifies tRNA genes without or with introns canonically located at position 37/38 in the anticodon-loop (Lowe and Eddy, 1997). This analysis identified a total of 30 predicted tRNA genes, which is insufficient to decode the 61 sense codons utilized in the nuclear genome of *C. merolae* (Matsuzaki et al., 2004). Therefore, to discover unidentified *C. merolae* nuclear tRNA genes, a genome-wide analysis was performed using the SPLITS and SPLITSX programs (Sugahara et al., 2006, 2007), which were developed to detect tRNA genes harboring BHB motifs, including *cis*-spliced tRNAs with introns inserted at various positions and split tRNAs that are joined at several positions in the cloverleaf structure. In addition, a BLAST search of conserved sequences in the TΨC-arm or anticodon-arm was also performed. This approach identified a total of 43 tRNA genes for 42 anticodons, which is sufficient to decode the 61 codons (Soma et al., 2007, 2013).

**Figure 1:**
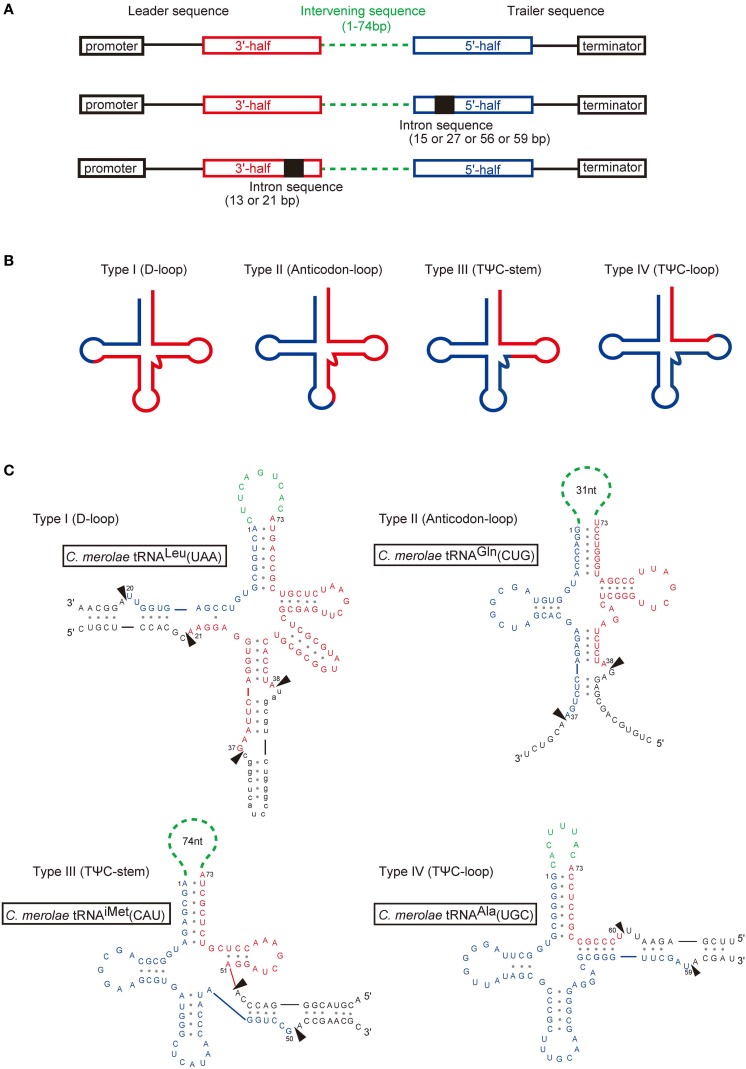
**Gene organization and structures of permuted tRNAs. (A)** Schematic representations of the structures of permuted tRNA genes with or without an intron. The 5′-half (blue) and the 3′-half (red) of the mature tRNA, the intron sequence (black), and the intervening sequence (green) of the pre-tRNA are shown. **(B)** Most permuted tRNAs can be classified into four types based on the location of the junction between the 3′-end of the 5′-half (blue) and the 5′-end of the 3′-half (red) in the secondary structure. **(C)** Inferred secondary structures of pre-tRNAs representing the four types of permuted tRNA genes in *C. merolae*. The arrowheads indicate the positions to be processed. The intron sequence is shown in lower case. The tRNA positions are numbered according to Marck and Grosjean (2002). The figures are partially identical to the Figure 1 of Soma et al. (2007).

Notably, for 11 of the 43 tRNA genes identified in *C. merolae*, the sequence encoding the 3′-half of the tRNA is positioned upstream of the sequence encoding the 5′-half in the genome (Figure 1A), and the two halves are interrupted by an intervening sequence that corresponds to the boundary connecting the 5′- and 3′-ends of the acceptor-stem of a mature tRNA. This arrangement is termed the circular gene permutation model (Heinonen et al., 1987; Pan et al., 1991; Keiler et al., 2000); hence, these genes were named “circularly permuted tRNA genes” (Soma et al., 2007). The study by Soma et al. (2007) was the first report of the existence of permuted genes encoding tRNAs or eukaryal nuclear-encoded non-coding RNAs. A TATA-like sequence was identified within the region 50 bp upstream of the 3′-half of most of the permuted tRNA genes as well as the non-permuted tRNA genes in *C. merolae* (Matsuzaki et al., 2004; Soma et al., 2013), indicating its importance for the transcription of tRNA genes. A T-stretch corresponding to a termination signal for RNA polymerase III (RNAPIII) was identified downstream of the 5′-half of these genes (Sprague, 1995; Hamada et al., 2000; Nielsen et al., 2013), but no promoter or termination signals were identified in the intervening region between the 3′- and 5′-halves, which varies in length from 7 to 74 nucleotides (Table 1). These observations suggest that the 3′- and 5′-halves of the putative tRNA genes are transcribed as a linear RNA. The exon sequences of both the permuted and non-permuted *C. merolae* tRNA genes show ordinary characteristics of eukaryal tRNAs and contain consensus elements found in eukaryotic tRNAs (Marck and Grosjean, 2002; Jühling et al., 2009), including U8, the R15:Y48 tertiary base pairing, G18G19, and U33; in addition, the U54U55C56 for elongator tRNAs and the A54U55C56 for initiator tRNA^Met^ are also conserved. The 3′-terminal CCA sequence, to which an amino acid is conjugated, is not encoded in the *C. merolae* genome, as found in other eukaryotes.

**Table 1:** **Characteristics of permuted tRNA genes in unicellular algae and archaea**.

**Organism**	**Permuted tRNA (anticodon)**	**Type (junction)**	**Intervening seq. (bp)**	**Intron (BHB, position, bp)**
**RED ALGA**				
*C. merolae*	Leu(TAA)	I (20/21)	10	hBHBh', 37/38, 21
	Gln(CTG)	II (37/38)	31	No
	Leu(CAG)	II (37/38)	9	No
	Thr(CGT)	II (37/38)	31	No
	Thr(AGT)	II (37/38)	24	No
	Gly(CCC)	II (37/38)	7	no h, 55/56, 13
	Gly(CCC)	II (37/38)	7	no h, 55/56, 13
	iMet(CAT)	III (50/51)	74	No
	Ala(TGC)	IV (59/60)	8	No
	Arg(CCT)	IV (59/60)	23	hBHBh', 14/15, 27
	Lys(TTT)	IV (59/60)	38	No
**GREEN ALGA**				
*O. lucimarinus*	Leu (TAA)	II (37/38)	25	No
	Ser(CGA)	II (37/38)	12	No
	Ser(TGA)	II (37/38)	13	no H, 27/28, 56
	Cys(GCA)	II (37/38)	26	No
*O. tauri*	Ser (CGA)^*^	V (15/16)	17	No
	Ser(CGA)	II (37/38)	28	No
	Ser(TGA)	II (37/38)	33	No
	Arg(TCT)	II (37/38)	11	No
	Asn(GTT)	II (37/38)	22	No
	Cys(GCA)	II (37/38)	38	No
*M. pusilla*	Ser (GCT)	II (37/38)	15	No
	Ser(CGA)	II (37/38)	19	No
	Ser(AGA)	II (37/38)	64	No
	iMet(CAT)	II (37/38)	47	No
	Leu(TAA)	II (37/38)	20	No
	Cys(GCA)	II (37/38)	12	No
*M.sp RCC299*	Ser (CGA)	II (37/38)	16	No
	Ser(TGA)	II (37/38)	12	No
	iMet(CAT)	II (37/38)	12	No
**CHLORARACHNIOPHYTE ALGA**				
*B. natans*	Ser (AGA)	II (37/38)	5	No
(nucleomorph)	Ser(CGA)	II (37/38)	5	No
**CRENARCHAEA**				
*T. pendens*	iMet (CAT)	IV (59/60)	7	no H, 37/38, 15
	Tyr(GTA)	IV (59/60)	1	no H, 37/38, 59

*Each gene is identified by the amino acid with which the tRNA is charged. Classification of permuted tRNAs (types I–V) is based on the location of the junction between the 5 ′-half and the 3 ′-half in the inferred secondary structure for pre-tRNAs. The length (bp, base pair) of sequence encoding for the intervening sequence and the presence of an intron are indicated. In C. merolae, two distinct genes that produce identical mature tRNA sequences encode tRNA^Gly^(CCC). iMet and eMet mean initiator and elongator tRNA^Met^(CAT), respectively. The asterisk indicates that the sequence of this tRNA is a typical, and experimental analysis would be preferable*.

As shown in Figure 1B, *C. merolae* permuted tRNA genes can be classified into four types (I–IV) based on the location of the junction between the 3′-end of the 5′-half and the 5′-end of the 3′-half in the inferred secondary structures of the pre-tRNAs. The junctions are located at position 20/21 in the D-loop (type I), position 37/38 in the anticodon-loop (type II), position 50/51 in the TΨ C-stem (type III), or position 59/60 in the TΨ C-loop (type IV). In *C. merolae*, one type I, six type II, one type III, and three type IV candidate tRNAs have been identified (Figure 1C, Table 1). The sequences adjacent to the junctions in the pre-tRNAs are predicted to form a BHB motif that is generally found around the intron-exon junctions of nuclear and archaeal pre-tRNAs (Figure 1C).

**Figure 2:**
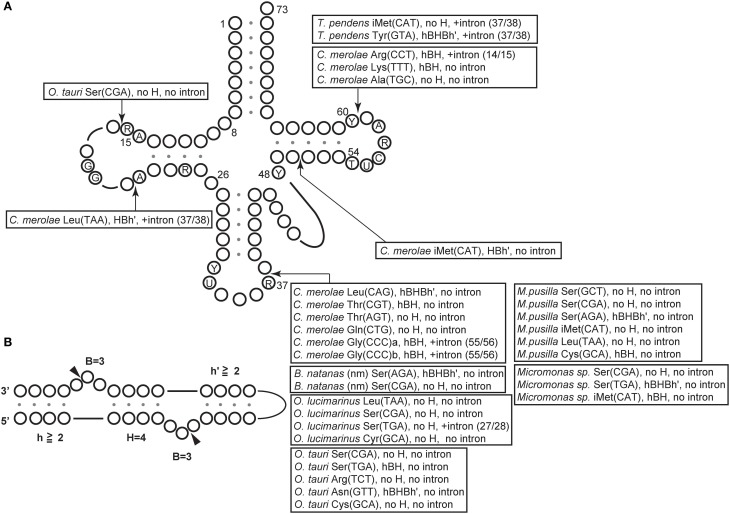
**Distribution of BHB motifs at the junctions of permuted tRNAs in algae and archaea. (A)** The tRNA nucleotides are numbered according to Marck and Grosjean (2002) and the arrows indicate the positions of the BHB motifs. The type of BHB motif and the presence or absence of an intron is indicated for each tRNA. For example, “*C. merolae* Leu(TAA), HBh', +intron, (37/38)” at the position between nucleotides 20 and 21 means that the HBh' is from the junction of the *C. merolae* permuted tRNA^Leu^(UAA), which contains an intron at 37/38. **(B)** The BHB motif is classified by one or two 3-nucleotide (nt) bulges (denoted as “*B* = 3”) separated by a central 4-base pair (bp) helix (denoted as “*H* = 4”) and flanked by two helices (denoted as h or h′), each with more than two base pairings. “hBHBh′” is the strict form of the BHB motif, which contains two bulges, a central helix and two flanking helices. The relaxed form of the BHB motif (BHL) lacking some of these bulges or helices is denoted as “hBH” or “HBh'.” The term “no H” represents motifs that do not contain a central 4-bp helix.

To date, 34 permuted tRNA genes have been identified in unicellular algae and archaea (Table 1), including 11 genes from the nuclear genome of the red alga *C. merolae* (Soma et al., 2007); 19 genes from the nuclear genome of four green algae (*Ostreococcus lucimarimus*, *Ostreococcus tauri*, *Micromonas Pusilla*, and *Micromonas* sp. *RCC299*) (Maruyama et al., 2010); two genes from the nucleomorph genome of the chlorarachiniophyte alga *Bigelowiella natans* (Maruyama et al., 2010), which is a remnant of a green algal nuclear DNA that developed as a secondary endosymbiont (Douglas et al., 2001; Archibald, 2007; Archibald and Lane, 2009); and two genes from the genome of the crenarchaeon *Thermofilum pendens* (Chan et al., 2011). In the nucleomorph and the nucleus of green algae, the junctions of the permuted pre-tRNAs are located most commonly at position 37/38 in the anticodon-loop (type II), while they are located at position 59/60 in the TΨ C-loop (type IV) in archaea (Figure 2A, Table 1). This tendency contrasts with that in the red alga *C. merolae*, in which the junctions are found at various positions in the cloverleaf structure. The intervening sequence varies from 1 to 74 bp among organisms (Table 1); tRNA^Tyr^(GTA) from *T. pendens* contains the shortest intervening sequence, while tRNA^iMet^(CAT) from *C. merolae* contains the longest intervening sequence. The species of amino acid or anticodon in the tRNAs encoded by permuted genes are not conserved among organisms. Interestingly, permuted tRNA^iMet^(CAT) exists in each lineage of red algae, green algae, and crenarchaea. In addition, tRNA^Ser^, tRNA^Leu^, and tRNA^Tyr^, which are classified as class II tRNAs and have long variable-arms (Rich and Rajbhandary, 1976; Dirheimer et al., 1995), tend to be encoded as permuted genes. This observation may imply that the evolution of the long variable-arm, which is the dominant element required for recognition by corresponding aminoacyl-tRNA synthetases (Asahara et al., 1993; Himeno et al., 1997a; Soma et al., 1999), is correlated with that of the tRNA gene structure. Indeed, the long variable-arm is suggested to have arisen from an intron (Kjems et al., 1989). Further analyses of the sequences of disrupted tRNAs will aid identification of the types of tRNA genes that tend to be permuted.

## Transcription of permuted tRNA genes

Northern blotting and aminoacylation analyses of *C. merolae* total RNA verified that tRNA molecules expressed from permuted genes are aminoacylated and are thus likely to participate in protein synthesis (Soma et al., 2007). Expression of some permuted tRNA genes from the nucleomorph and the nucleus of green algae has also been confirmed by northern blotting or reverse transcription polymerase chain reaction analyses (Maruyama et al., 2010). However, the function of mature tRNAs in the nucleomorph is unclear because protein synthesis in these structures has not yet been observed experimentally (Archibald and Lane, 2009; Curtis et al., 2012). The two permuted tRNAs in the archaea *T. pendens* are both encoded by single-copy genes for a unique anticodon that cannot be supplemented by other isoacceptors (Chan et al., 2011); therefore, they must be expressed and produce functional tRNA molecules.

The identification of unusual (permuted and atypical intron-containing) tRNA genes in eukaryotes raised an intriguing question about the mechanism of transcription. In eukaryotes, transcription of tRNA genes is generally performed by RNAPIII and it relies on an intragenic bipartite promoter consisting of an A box and a B box, which correspond to the highly conserved sequences in the D-arm (positions 8–19) and TΨ C-arm (positions 52–62), respectively, (Galli et al., 1981; Ciliberto et al., 1983; Sprague, 1995; Guffanti et al., 2006; Marck et al., 2006). The protein factors that bind to these motifs have been well-characterized in yeast (Willis, 1993; Paule and White, 2000; Geiduschek and Kassavetis, 2001; Huang and Maraia, 2001; Schramm and Hernandez, 2002). Polymerase III C (TFIIIC), a multi-subunit complex of transcription factors that is essential for transcription by RNAPIII, binds to the A and B boxes simultaneously and promotes binding of the TFIIIB complex, which includes the TATA-box binding protein, to the region upstream of the tRNA sequence, followed by recruitment of RNAPIII. The dependency of transcription of tRNA genes on the A and B boxes is predominantly conserved; however, the additional requirements for transcription are diverse among organisms and the upstream region sometimes contributes to the efficiency of the initiation step (Choisne et al., 1998; Yukawa et al., 2000; Hamada et al., 2001; Giuliodori et al., 2003; Dieci et al., 2006).

In permuted tRNA genes, the A box and B box are located inversely and are interrupted by an intervening sequence of variable length. This positional relationship is unsuitable for TFIIIC binding; therefore, the A and B boxes may not be uniformly bound by TFIIIC and the intragenic promoter may be dispensable for transcription of these genes. Instead, an upstream TATA-like sequence and a downstream T-stretch, which are probably the promoter and termination signal, respectively, (Sprague, 1995; Hamada et al., 2000, 2001; Nielsen et al., 2013), are located close to most permuted tRNA genes in *C. merolae* (Soma et al., 2007). This genomic arrangement also occurs for non-permuted tRNA genes in *C. merolae*, and the A and B boxes in the promoters of these genes may not be recognized by TFIIIC because they are often interrupted by a single or multiple (up to three) introns of various lengths (11–69 bp) (Matsuzaki et al., 2004; Soma et al., 2013). Homologs of TFC1 and TFC3, the TFIIIC components that are responsible for binding to the A and B boxes, have not been identified in *C. merolae* (Matsuzaki et al., 2004; Nozaki et al., 2007). Taken together, these findings suggest that *C. merolae* employs a non-canonical transcription system that is independent of TFIIIC and directs recruitment of TFIIIB to the upstream TATA-box, thereby enabling the transcription of various types of tRNA genes. An ambiguous AT-rich region is also located upstream of some permuted tRNA-encoding sequences in the *B. natans* nucleomorph and the nucleus of green algae (Maruyama et al., 2010). Therefore, TATA-like sequence-dependent transcription of tRNA genes may predominate in algae. This possibility is supported by the fact that an upstream TATA box is well conserved and functionally important for transcription of tRNA genes in some plants and fungi (Choisne et al., 1997, 1998; Yukawa et al., 2000; Hamada et al., 2001; Dieci et al., 2006). In addition, transcription of *Saccharomyces cerevisiae* tRNA genes harboring an upstream TATA box proceeds without TFIIIC *in vitro* (Dieci et al., 2000).

In archaea, transcription of a stable RNA depends on the upstream promoter including BRE (TFB response element) and TATA box (Wich et al., 1986; Thomm and Wich, 1988; Palmer and Daniels, 1995; Reeve, 2003), and on a downstream poly T sequence, which contributes to transcription termination (Santangelo et al., 2009). In *T. pendens*, which harbors two permuted tRNA genes, a predicted AT-rich promoter is located upstream of most of its tRNA genes (Chan et al., 2011), suggesting that various types of tRNA genes are potentially expressed. Consistent with this notion, *T. pendens* contains a large number of tRNA genes that are disrupted by various introns (Sugahara et al., 2009; Chan et al., 2011; Fujishima et al., 2011).

## Maturation of permuted pre-tRNAs via a circular RNA intermediate

**Figure 3:**
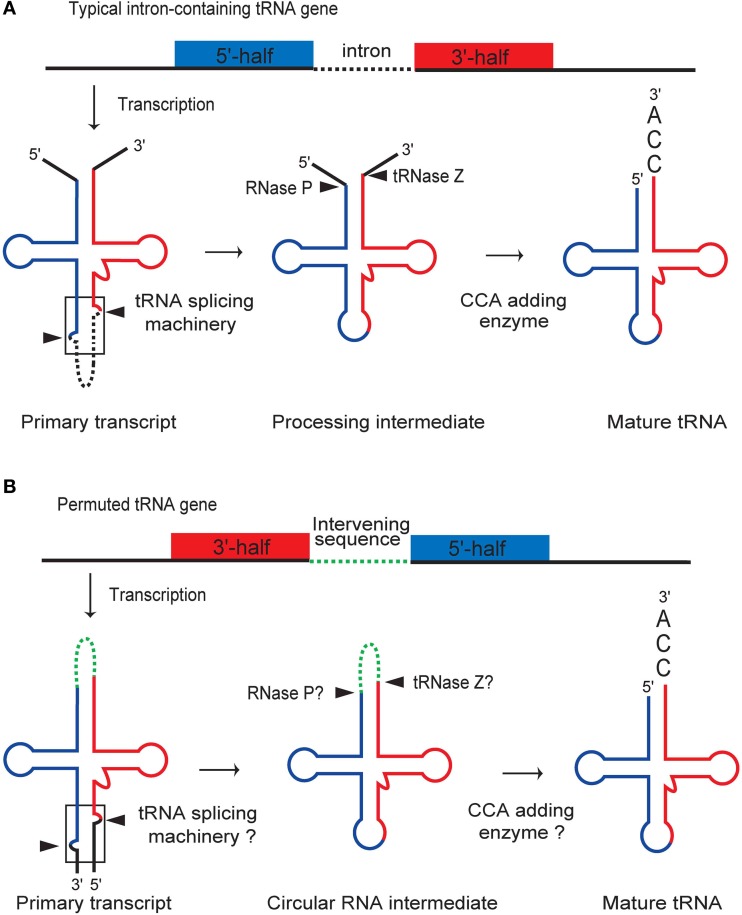
**Comparison of the processing pathways for typical intronic and permuted pre-tRNAs. (A)** Maturation of a typical intronic pre-tRNA involves intronic splicing, processing of the 5′- and 3′-ends by RNase P and tRNAse Z, and addition of the 3′-terminal CCA sequence. **(B)** Maturation of a permuted pre-tRNA starts with processing of the BHB motif (boxed) by the tRNA-splicing machinery, resulting in the formation of a circular RNA intermediate. The intervening sequence is then removed by RNase P and tRNase Z, followed by CCA addition. The sequential processing of permuted pre-tRNAs in the proposed pathway may be accomplished using processing machineries that are commonly used for typical pre-tRNAs, because the recognition elements for each processing enzyme are conserved in the permuted pre-tRNA and circular intermediate. The figure is partially identical to the Figure 3C of Soma et al. (2007).

Processing of a pre-tRNA typically involves intronic splicing, maturation of the 5′- and 3′-ends at the acceptor stem, and nucleotide modification (Figure 3A) (Deutscher, 1995; Hopper and Phizicky, 2003). Biochemical analyses have shown that permuted pre-tRNAs in unicellular algae are maturated by a processing pathway that utilizes a circular RNA intermediate to exchange the location of the 5′- and 3′-halves of the tRNA (Soma et al., 2007, 2013; Maruyama et al., 2010). Reverse transcription polymerase chain reaction and sequencing analyses identified the following processing intermediates derived from algal permuted tRNAs: a circularly permuted pre-tRNA, the sequence of which aligns in the order of the leader sequence, the 3′-half of tRNA, the intervening sequence, the 5′-half of tRNA, and then the trailer sequence; and a circular RNA intermediate, in which the leader and trailer sequences are removed and the resulting ends are ligated, while the intervening sequence is retained. Furthermore, a consistent PCR product was also observed in these analyses, suggesting that two rounds of reverse transcription occur around a circular intermediate, thereby confirming the presence of the circular RNA molecule. Terminal sequences were also verified for a mature tRNA, in which the extra sequences are removed and the CCA sequence is added post-transcriptionally to the 3′-terminus of the acceptor-stem, as occurs in other eukaryotes.

As summarized in the model presented in Figure 3B, maturation of permuted pre-tRNAs in algal cells probably starts with processing of the junction of the termini to form a circular RNA intermediate in which the termini are joined by the intervening sequence. The intervening sequence in the acceptor-loop of the circular RNA intermediate is then removed, possibly by RNase P (Altman et al., 1995; Jarrous and Gopalan, 2010; Altman, 2011) and tRNase Z (Deutscher, 1995; Schürer et al., 2001; Schiffer et al., 2002; Späth et al., 2007), which are universal endoribonucleases. Finally, the 3′-terminal CCA sequence is added (Weiner, 2004) to generate the functional acceptor-stem of the tRNA. Because the circular RNA intermediate has been detected in red and green algae, this model is likely to be common to permuted tRNAs of both types of algae.

Cleavage of the leader and trailer sequences at the junction of permuted pre-tRNAs is most likely performed by the tRNA-intron splicing machinery, because the sequences adjoining the processing sites potentially form a BHB motif, which is the dominant recognition element for nuclear and archaeal tRNA-splicing endonucleases (Figures 1C, 2B). After excision of the BHB motifs at the junction, subsequent ligation of the 5′- and 3′-termini of the exons is required and is probably carried out by tRNA-splicing ligase (Xu et al., 1990b; Westaway and Abelson, 1995; Englert et al., 2011, 2012; Popow et al., 2011). It is intriguing that various positions in the cloverleaf structure of tRNAs, even the core region of the L-shaped tertiary structure, can serve as termini for permuted pre-RNA molecules that are recognized by the splicing machinery.

RNase P (McClain et al., 1987; Christian et al., 2002; Zahler et al., 2003; Kirsebom, 2007; Reiter et al., 2010; Altman, 2011) and tRNase Z (Nashimoto et al., 1999; Li de la Sierra-Gallay et al., 2006; Späth et al., 2007; Minagawa et al., 2008) generally recognize the top half of the L-shaped tertiary structure of a tRNA corresponding to the acceptor-stem and the TΨ C-arm, and do not require the mature body of the tRNA. Therefore, these enzymes may also perform endonucleolytic cleavage of the acceptor-loop of a circular RNA intermediate. Although some endoribonucleases require the linear ends of substrates to function (Mackie, 1998; Suzuki et al., 2006), it is not known whether this condition holds for RNase P and tRNase Z. The intron in the D- and TΨ C-arm, which inhibits folding of the tertiary structure of a tRNA, should be removed before processing of the acceptor-loop by RNase P and tRNase Z. Consistent with this requirement, the intron in the TΨ C-loop of a circular intermediate of *C. merolae* tRNA^Gly^, which harbors both intronic and permuted structures, is removed before the intervening sequence at the acceptor-loop is processed (Soma et al., 2013). This finding can be explained by the fact that the top half of substrates for *C. merolae* tRNase Z must form a canonical tertiary structure, and circular pre-tRNAs without an intron would be able to fold into the canonical tertiary structure, which agrees with the previous discovery that artificially permuted tRNA molecules can fold into correct tertiary structures (Pan et al., 1991).

In eukaryotes, each tRNA processing step occurs at a different location in the cell, and the cellular distribution of processing enzymes is not conserved among organisms. In animal cells, the tRNA-splicing endonuclease and ligase are localized to the nucleus (Westaway and Abelson, 1995; Paushkin et al., 2004). By contrast, in budding yeast, the endonuclease is present on the surface of mitochondria (Huh et al., 2003; Yoshihisa et al., 2003) and the ligase is present in the cytosol (Huh et al., 2003). RNase P and tRNase Z are found in the nucleus and/or cytoplasm in eukaryotic cells (Späth et al., 2007; Canino et al., 2009; Gobert et al., 2010; Pinker et al., 2013). Accordingly, the order of the processing steps of a permuted pre-tRNA in algal cells will likely be governed by the location of the enzymes required.

It is unclear whether maturation of archaeal permuted pre-tRNAs involves the formation of a circular RNA intermediate. In a recent study, an *in vitro* transcript simulating a permuted pre-tRNA, which was composed of a tandem repeat of intron-containing tRNA, was cleaved at the BHB motif by a recombinant splicing endonuclease from the euryarchaeon *Methanococcus jannaschii* (Tocchini-Valentini and Tocchini-Valentini, 2012), suggesting that archaeal permuted pre-tRNAs can be processed in a similar pathway to that found in algae. Analysis of permuted pre-tRNA processing in *T. pendens* may also help to clarify whether the physiological role of permuted tRNA genes is ascribed to the formation of the circular RNA intermediate. With the exception of *Nanoarchaeum equitans* (Randau et al., 2008; Heinemann et al., 2010), RNase P and tRNase Z generally contribute to the end maturation of tRNAs in archaea (Späth et al., 2007; Jarrous and Gopalan, 2010). The 3′-terminal CCA sequence of two permuted tRNAs is encoded in the genome sequence of *T. pendens* (Chan et al., 2011) and one of these genes contains a short intervening sequence of only one nucleotide. It will be intriguing to clarify how such a short intervening sequence in the acceptor-loop is removed.

## Processing of an intron in permuted pre-tRNAs

Four tRNA genes from the red alga *C. merolae* (Soma et al., 2007), one tRNA gene from the green alga *O. lucimarinus* (Maruyama et al., 2010), and two tRNAs genes from the crenarchaeon *T. pendens* (Chan et al., 2011) contain an intron in the 5′- or 3′-half of the gene (Figure 1A, Table 1), meaning that their pre-tRNAs require splicing of an intron in addition to swapping of the 5′- and 3′-halves. The position of the intron is not conserved among these organisms; in the four *C. merolae* tRNA genes, the introns are inserted at various positions (the D-loop, the anticodon-loop, and the TΨ C-loop), while those in the *O. lucimarinus* and *T. pendens* tRNA genes are inserted at specific positions: 27/28 in the anticodon-stem and 37/38 in the anticodon-loop, respectively. In *C. merolae* and *T. pendens*, the intron-exon junction and the termini of permuted pre-tRNAs harboring an intron can each form an independent BHB motif. The two BHB motifs are not nested; therefore, processing of one BHB motif can be preceded by processing of the other. Using *C. merolae* tRNA^Gly^(CCC), which possesses both permuted (with the junction at position 37/38 in the anticodon-loop) and intronic (inserted at position 55/56 in the TΨ C-loop) structures, it was determined that the BHB motif in the intron is processed before the BHB motif in the termini of permuted pre-tRNA^Gly^(CCC) (Soma et al., 2013). The theoretical ΔG of the BHB motif in the intron was calculated to be slightly lower than that of the BHB motif in the termini. The same phenomenon was also observed for precursors transcribed from multiple intron-containing (but not permuted) tRNA genes in *C. merolae*, in which the BHB motifs in the pre-tRNAs were removed in the order dictated by the theoretical free energy of each motif (Soma et al., 2013). These findings indicate that multiple BHB motifs in permuted and/or intronic pre-tRNAs in *C. merolae* are processed sequentially, even when each BHB motif can fold independently. This feature may be attributable to the stability of each BHB motif and their accessibility to the splicing endonuclease. Alternatively, it may depend on the position of the BHB motifs, because the BHB motif at the canonical position 37/38 is always the final substrate and has a relatively high ΔG. BHB motifs at 37/38, even those that form the junction of the permuted pre-tRNA or the intron, may be recognized by *C. merolae* endonuclease only after BHB motifs at the other positions have been processed. This procedure contrasts with the processing of multimeric introns in some archaeal pre-tRNAs, in which the introns are nested and the last intron can form a BHB motif only after the other introns are processed (Sugahara et al., 2007; Tocchini-Valentini et al., 2009).

## Correlation between the BHB motif at the junction of permuted pre-tRNAs and the substrate specificity of splicing endonucleases

**Figure 4:**
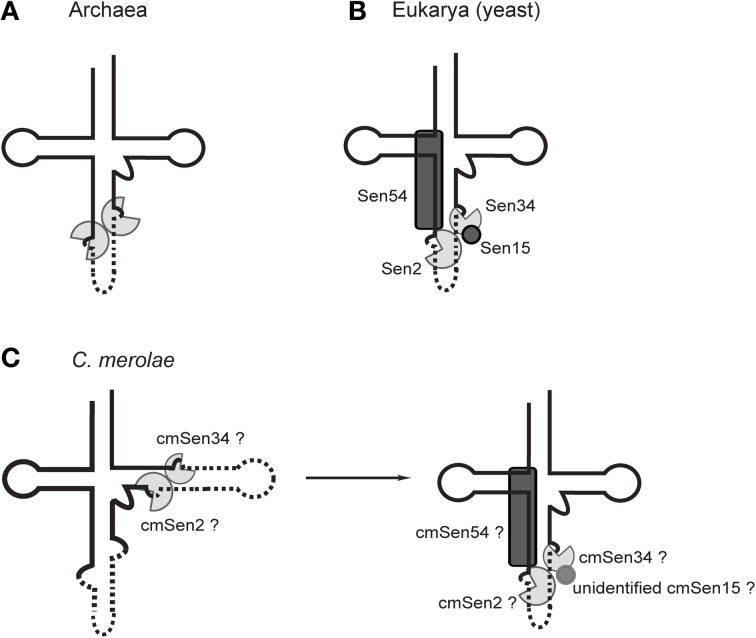
**Schematic representations of the tRNA-splicing endonuclease in archaea and eukaryotes. (A)** An archaeal dimeric endonuclease comprising two catalytic subunits. **(B)** The yeast heterotetrameric endonuclease comprising two catalytic subunits (Sen2 and Sen34) and two accessory subunits (Sen15 and Sen54). **(C)** The possible heterodimeric (cmSen2 and cmSen34), heterotrimeric (cmSen2, cmSen34, and cmSen54), and heterotetrameric (cmSen2, cmSen34, cmSen54, and unidentified cmSen15) forms of the *C. merolae* endonuclease.

The BHB motif is the dominant recognition element for all known nuclear and archaeal tRNA-splicing endonucleases (Fruscoloni et al., 2001; Marck and Grosjean, 2003; Tocchini-Valentini et al., 2005a; Xue et al., 2006; Calvin and Li, 2008) and processing by these enzymes should have been pivotal for the development and maintenance of permuted tRNA genes in the genome. Archaeal endonucleases exhibit symmetrical architectures, and recognition of the splice sites of intronic pre-tRNAs by these enzymes is largely dependent on the BHB motif (Figure 4A) (Thompson and Daniels, 1988; Diener and Moore, 1998; Tocchini-Valentini et al., 2005a,b; Calvin and Li, 2008). In most archaeal tRNAs, the BHB motifs develop a relaxed form (hBH, as shown in Figure 2B) and are located at position 37/38 in the anticodon-loop, while several species from Crenarchaeota contain introns at non-canonical positions, such as the anticodon-arm, D-arm, TΨ C-arm, variable-arm, or acceptor-stem, with strict (hBHBh') or relaxed (hBH, BHh', or no H) forms of the BHB motif (Marck and Grosjean, 2003; Tocchini-Valentini et al., 2005a; Sugahara et al., 2007, 2008, 2009). In addition to tRNAs, BHB-mediated introns are also found in rRNAs and mRNAs in some archaea (Kjems and Garrett, 1988; Tang et al., 2002; Watanabe et al., 2002; Yoshinari et al., 2006). Furthermore, the combination of RNA fragments during maturation of split tRNAs depends on the processing of the BHB motifs by the tRNA-splicing machinery in *N. equitans* (Randau et al., 2005c), indicating that BHB-mediated disruption of genetic information and its processing by splicing endonucleases is widespread in archaea. Four different types of endonuclease have been identified in archaea (Tocchini-Valentini et al., 2005a; Calvin and Li, 2008; Fujishima et al., 2011; Hirata et al., 2011); the subunit architecture of these endonucleases seems to have co-evolved, by “subfunctionalization,” with their substrate specificity (Tocchini-Valentini et al., 2005b, 2007). *T. pendens* contains a heterotetrameric endonuclease (α2β 2) that can recognize both strict (hBHBh') and relaxed (BHL) motifs, and the junction of its two permuted tRNAs comprises no H or hBHBh' motif, and is located at position 59/60 in the TΨ C-loop (Figure 2, Table 1). The broad substrate specificity of the *T. pendens* endonuclease would have allowed the development and maintenance of permuted tRNAs during evolution.

The *S. cerevisiae* splicing endonuclease forms a heterotetrameric structure (αβδε) comprised of two catalytic subunits (Sen2 and Sen34) and two accessory subunits (Sen15 and Sen54) (Rauhut et al., 1990; Westaway and Abelson, 1995; Trotta et al., 1997; Calvin and Li, 2008). Interactions between Sen2 and Sen54, and between Sen15 and Sen34, were identified by a yeast two-hybrid experiment (Trotta et al., 1997). These four subunits function cooperatively to recognize cleavage sites via “a ruler mechanism,” in which the endonuclease measures a specified distance to the site at which the cuts should be made in a pre-tRNA (Figure 4B) (Greer et al., 1987; Reyes and Abelson, 1988; Westaway and Abelson, 1995; Fabbri et al., 1998; Calvin and Li, 2008). In addition to the typical hBH motif at the canonical 37/38 position, yeast endonuclease recognizes the mature domain of pre-tRNA and the base pairs between the anticodon and the intron (A·I base pairs) (Mattoccia et al., 1988; Baldi et al., 1983, 1992; Di Nicola Negri et al., 1997; Trotta et al., 2006; Xue et al., 2006). Similarly, wheat germ endonuclease recognizes some specific nucleotides in the D-stem, and the mature tRNA domain is required for adequate binding to the endonuclease (Stange et al., 1992). Coordination between all four subunits of eukaryal endonucleases would stabilize the enzyme to place its active site at a specific position in the cloverleaf structure of pre-tRNA, namely position 37/38. Thus, it is likely that the recognition system and asymmetric subunit architecture of eukaryal endonucleases have co-evolved strictly with the BHB motifs at position 37/38.

The junctions of permuted pre-tRNAs in the *B. natans* nucleomorph and the nucleus of green algae comprise a hBH motif and are located at position 37/38 in the anticodon-loop (type II), which are the characteristics for recognition by the eukaryal splicing endonuclease (Table 1, Figure 2). In addition, *B. natans* and green algae contain almost no tRNA genes harboring atypical introns (Palenik et al., 2007; Maruyama et al., 2010). On the contrary, in the red alga *C. merolae*, the junctions of permuted pre-tRNAs and introns comprise various types of BHB motifs and are scattered along the cloverleaf structure (Figure 2A). This arrangement suggests that the *C. merolae* splicing endonuclease recognizes a wide variety of BHB motifs and employs a recognition strategy that is different from that of the known eukaryotic endonucleases.

A search of the *C. merolae* genome identified homologs of three of the yeast endonuclease subunits (cmSen2, cmSen34, and cmSen54) (Soma et al., 2013); however, no apparent homolog of the Sen15 accessory subunit was identified by homology searching or yeast two-hybrid analyses, which conflicts with the notion that all four subunits are essential for functional multimerization of the endonuclease. In yeast, Sen15 interacts with Sen34 to aid the proper positioning of the 3′-splice site (Westaway and Abelson, 1995; Di Nicola Negri et al., 1997; Trotta et al., 1997; Fabbri et al., 1998; Xue et al., 2006). The *C. merolae* endonuclease may contain an unidentified subunit or may comprise a novel heterotrimeric complex (Figure 4C). However, the *C. merolae* endonuclease containing accessory subunits is not likely to interact with pre-tRNAs that are disrupted at positions other than 37/38, because yeast Sen54 probably interacts with the D-arm and the acceptor-stem that are located in the core region of the L-shaped tertiary structure of a pre-tRNA (Di Nicola Negri et al., 1997; Xue et al., 2006). Thus, the *C. merolae* endonuclease may act on these pre-tRNAs as a dimer composed of catalytic subunits only (cmSen2 and cmSen34), via a tRNA mature domain-independent recognition mechanism. It is also tempting to speculate that the subunit composition of the *C. merolae* endonuclease depends on the positions or types of BHB motifs in the substrates. A feasible model may be that BHB motifs at positions other than 37/38 are removed by cmSen2-cmSen34, making the BHB motif at position 37/38 accessible to cmSen2-cmSen54-cmSen34 or cmSen2-cmSen54-cmSen34-cmSen15(unidentified), which interacts with the mature domain of the pre-tRNA, as occurs in yeast (Figure 4C). A previous study showing that the BHB motif at the canonical 37/38 position is always the final substrate during tRNA processing in *C. merolae* cells (Soma et al., 2013) may support this hypothesis. These observations imply that processing of the BHB motif in eukaryal tRNAs is more divergent among species than previously thought. Various types of BHB-mediated disrupted tRNA genes and splicing endonucleases may be present in other eukaryotes. In fact, ectopic intron-containing tRNA genes have been discovered in the nucleomorph of the cryptomonad *Guillardia theta* (Kawach et al., 2005), although many of these introns do not form a defined BHB motif. Furthermore, the absence of an accessory subunit (Sen15) homolog in *Arabidopsis thaliana* (Akama et al., 2000) implies that plant endonucleases have evolved various patterns of subunit architectures. On the other hand, *A. thaliana* contains only a few species of canonical intron-containing tRNA genes and does not contain any other disrupted tRNA genes; therefore, its endonuclease has not been adapted to process non-canonically disrupted pre-tRNAs.

## Implications for the physiological relevance of permuted tRNA genes

To date, circular gene permutation of non-coding RNAs other than tRNA has been reported for the LSU rRNA from *Tetrahymena* mitochondria (Heinonen et al., 1987) as well as bacterial and organellar tmRNAs (Keiler et al., 2000; Mao et al., 2009), the latter of which are involved in the *trans*-translation system that rescues stalled ribosomes and maintains quality control of proteins in the cell (Keiler et al., 1996; Himeno et al., 1997b; Muto et al., 1998). However, permuted tRNAs show some substantial differences to permuted rRNAs and tmRNAs. A pre-tRNA of a permuted tRNA gene is processed and re-ligated at the junction of the 5′- and 3′-halves. The resultant tRNA molecule is composed of a continuous single-stranded RNA that can form a canonical cloverleaf structure, which is equipped with a functional acceptor-stem and an anticodon in the proper position. By contrast, the corresponding breaks between the 5′- and 3′-halves of rRNAs and tmRNAs encoded by permuted genes are not ligated and they function in a two-piece form. In the case of tmRNA, this form has been suggested to have a beneficial function, perhaps by solving topological problems on the ribosome (Williams, 2002; Sharkady and Williams, 2004). This idea is supported by the independent evolution of a similar two-piece form of tmRNA, encoded as a permuted gene in different lineages of bacteria (Sharkady and Williams, 2004; Williams, 2004). Additionally, the location of the junction of the 5′- and 3′-halves differs between permuted tmRNAs and permuted tRNAs. The two-piece form of tmRNA is adapted to its functional advantage, and the corresponding breakage between the 5′-half and the 3′-half is located at a unique position downstream of the tag peptide coding region. By contrast, the junctions of permuted tRNAs are located at various positions in the cloverleaf structure because breakage at any position is ultimately ligated to produce a typical tRNA molecule. Consequently, permutation of genes encoding tRNAs does not seem to affect the authentic function of the mature tRNA or confer any physiological benefit or restriction.

In *C. merolae*, disrupted tRNA genes that exhibit permuted (7/43), intron-containing (23/43), or both types of structures (4/43) account for 79.1% (34/43) of all nuclear tRNA genes (Soma et al., 2013), whereas only a few protein-encoding genes have spliceosomal introns (Matsuzaki et al., 2004). The conservation of a large number of permuted tRNAs, in addition to intronic tRNAs, which require more extensive processing, in the streamlined genome of *C. merolae*, implies that BHB-mediated disruption of tRNA genes has some physiological meaning. It is well known that while some tRNA introns are dispensable (Mori et al., 2011) others are involved in post-transcriptional modification (Johnson and Abelson, 1983; Szweykowska-Kulinska and Beier, 1992; Björk, 1995), quality control to ensure the supply of precisely processed tRNA molecules to the cytosol (Arts et al., 1998; Lund and Dahlberg, 1998; Takano et al., 2005; Hopper, 2013), and regulation of the cell cycle in response to DNA damage (Ghavidel et al., 2007; Weinert and Hopper, 2007). Therefore, permuted tRNA genes may contribute to essential cell functions. Alternatively, the circular RNA intermediate may be preferable because of its resistance to degradation in the cell.

From a physiological point of view, a possible explanation for the maintenance of disrupted tRNA genes is protection against mobile elements (Randau and Söll, 2008). Fragmentation of tRNA genes is thought to prevent the integration of mobile elements because tRNA gene sequences are sometimes used as conventional target sites in the genome (Devine and Boeke, 1996; Hani and Feldmann, 1998; Mou et al., 2006). This direct and valuable strategy would have functioned as a selective pressure at some point during evolution to increase the number of permuted tRNA genes. This possibility may be supported by the fact that almost no recognizable transposons or viruses are found in the contemporary genomes of *C. merolae* and *M. pusilla*, which harbor permuted tRNA genes (Matsuzaki et al., 2004; Worden et al., 2009). By contrast, *Ostreococcus* species, which contain some permuted tRNA genes and *cis*-spliced tRNA genes, have many transposons (Worden et al., 2009; Maruyama et al., 2010). Genome-wide analyses and studies focusing on the relationship between mobile elements and disrupted tRNA genes should further our understanding of this concept.

The eukaryal tRNA processing system has proofreading functions to ensure that only mature tRNAs are supplied for translation, and yeast cells possess multiple pathways to degrade inappropriately processed and folded tRNAs (Arts et al., 1998; Lund and Dahlberg, 1998; Kadaba et al., 2004; Takano et al., 2005; Whipple et al., 2011; Hopper, 2013; Kramer and Hopper, 2013). In *Xenopus laevis* oocytes, intron-containing pre-tRNAs are exported from the nucleus less efficiently than intron-spliced tRNAs, and nucleotide modifications and removal of the 5′- and 3′-flanking sequences at the acceptor-stem are monitored before transport of tRNAs into the cytosol (Arts et al., 1998). Therefore, the BHB motifs at various positions of permuted pre-tRNAs and the acceptor-loop of the circular RNA intermediate inhibit their exportin-dependent transport from the nucleus, and the sequential processing of permuted pre-tRNAs would contribute to the discrimination of immature tRNAs, providing a selective pressure to retain them in the genome. *C. merolae* cells use a small repertoire of tRNAs; hence, the quality of tRNA molecules must be checked to guarantee translational fidelity. Furthermore, elimination of incorrectly processed tRNA molecules might be more important for organisms that harbor a splicing endonuclease with relaxed substrate specificity.

A different perspective is that permuted tRNA genes might have been formed as a remnant of genome dynamics under relatively neutral selective pressure. Even if such tRNA genes were acquired, most of them could not be retained because of the failure of transcription or subsequent RNA processing. In some organisms, including early-rooted algae and archaea, permuted tRNA genes could have persisted in the genome because of the upstream promoter-dependent transcription system and the capacity of the splicing machinery to process disrupted pre-tRNAs into the canonical cloverleaf structure. An expression system adapted to the wide variety of tRNA genes might have been preferable for organisms attempting to reduce redundantly duplicated tRNA genes, thereby enabling disruption of tRNA genes in various ways while maintaining the repertoire of those essential for protein synthesis. It has been suggested that permuted tRNA genes might have contributed to the maintenance of genome integrity during the reduction of the *B. natans* nucleomorph genome, which is the smallest eukaryotic genome (Gilson et al., 2006; Maruyama et al., 2010). Thus, plasticity of tRNA gene structure and expression systems may be more important than permuted tRNA genes.

## Scenarios for the development of permuted tRNA genes

There are two hypotheses for the development of permuted tRNA genes: the “ancient origin” hypothesis, which is related to the origin of the cloverleaf structure of tRNA (Di Giulio, 2008; Fujishima et al., 2008); and the “recent origin” hypothesis, which assumes that permuted tRNA genes arose from existing tRNAs in a relatively late stage of evolution (Randau and Söll, 2008; Sugahara et al., 2008; Maruyama et al., 2010; Chan et al., 2011).

The cloverleaf structure of tRNA is thought to have originated from mini-hairpins (Weiner and Maizels, 1987; Di Giulio, 1992, 2006; Schimmel and Ribas De Pouplana, 1995; Widmann et al., 1995), and tRNA sequences sometimes form a double hairpin structure flanked by the anticodon sequence (Tanaka and Kikuchi, 2001). The dominant localization of introns at position 37/38, which divide tRNAs into two hairpins, may be a remnant of the boundary connecting the hairpins, and disrupted tRNAs may represent plesiomorphic forms produced during the development of the modern cloverleaf structure (Di Giulio, 2008; Fujishima et al., 2008). The results of archaeal genome analyses have consistently suggested that modern tRNAs evolved through the combination of 5′-half and 3′-half fragments (Fujishima et al., 2008). Based on this concept, it was proposed that permuted tRNA genes arose from an event in which the two hairpin-like structures encoding the 5′- and 3′-halves of a tRNA were brought together in an inverted configuration on the genome (Di Giulio, 2008). However, there is some debate surrounding this idea. First, some permuted tRNAs are intervened at positions other than 37/38, which conflicts with the assumptions of the hairpin model (Di Giulio, 2008). Second, it is questionable whether the ancient forms of tRNA genes are preserved in the modern genome (Randau and Söll, 2008).

Based on comparative genome analyses, another hypothesis suggests that BHB-mediated disrupted tRNA genes were gained by gene transfer as apomorphies or were developed from extant tRNA genes (Di Giulio, 2008; Randau and Söll, 2008; Sugahara et al., 2009, 2012; Fujishima et al., 2010; Maruyama et al., 2010; Chan et al., 2011). Given that permuted tRNAs are present in early-rooted algae (Nozaki et al., 2003, 2007; Matsuzaki et al., 2004) and deep-branching Crenarchaeota from which eukarya might have derived (Lake et al., 1984; Cox et al., 2008), the algal genome may retain permuted tRNAs as a vestigial trait inherited from archaea. In fact, *C. merolae* tRNAs exhibit some characteristics that are found in archaea but not eukaryotes. For example, a number of *C. merolae* tRNAs contain ectopic and multiple introns, and *C. merolae* tRNA^Ile^ has the anticodon GAU (Matsuzaki et al., 2004), which has been identified in prokaryotes but not eukaryotes. However, sequence and structural similarities of the disrupted tRNAs in *C. merolae* and archaea have not been identified. Moreover, archaeal permuted tRNA genes encode the terminal CCA sequence, which is not encoded in the eukaryal genome, indicating that they have not simply been exchanged between archaea and algae (Chan et al., 2011). Therefore, permuted tRNA genes might have arisen independently in each lineage. This possibility is supported by the fact that BHB-mediated disrupted tRNA genes exhibit a discontinuous and patchy distribution in eukaryotes and archaea (Maruyama et al., 2010; Chan et al., 2011; Soma et al., 2013). An evolutionary relationship between *cis*-spliced tRNAs and split tRNAs has been suggested, because the leader sequences of some split tRNAs show a high degree of homology to the intronic sequence of tRNAs in correlated archaea (Fujishima et al., 2010). In addition, continuous transcripts corresponding to read-through of adjacently encoded 5′- and 3′-halves of split tRNAs are produced, albeit at very low levels, suggesting that they represent a transition state between a split tRNA and a *cis*-spliced tRNA in the genome (Chan et al., 2011). Thus, it is possible that permuted tRNAs emerged from extant tRNA genes.

**Figure 5:**
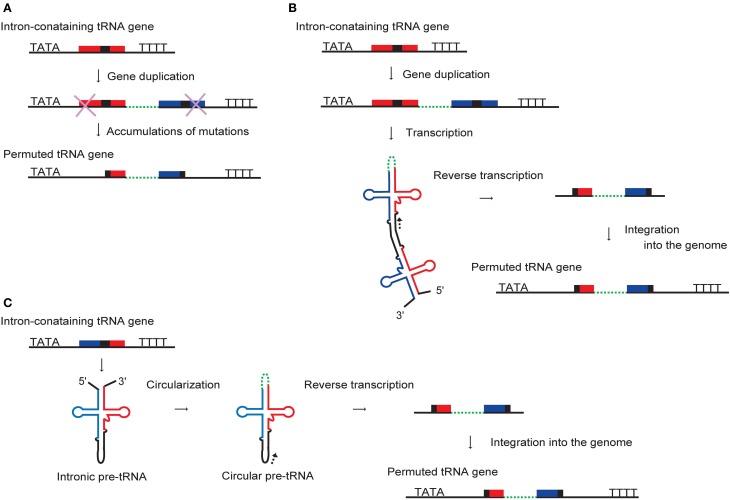
**Proposed models for the development of permuted tRNA genes**. **(A)** Permuted tRNA genes might have formed by gene duplication and loss of the outer segments. A tandem repeat of an intron-containing tRNA gene could be arranged into a permuted tRNA gene by the accumulation of mutations at both ends. Alternative models are based on reverse transcription of permuted or circularized pre-tRNA molecules. **(B)** A permuted pre-tRNA formed by an interaction between the 3′-half of the tRNA encoded by the initial gene and the 5′-half of the duplicated tRNA derived from the tandemly-repeated gene may have been reverse transcribed from the point indicated by an arrowhead and integrated back into the genome. **(C)** A circular intron-containing pre-tRNA produced by ligation of an intron-containing pre-tRNA at the acceptor-stem may also have been reverse transcribed and integrated into the genome.

A plausible description of the emergence of permuted tRNA genes via convergent evolution can be inferred from the model proposed for permuted rRNAs and tmRNAs, which function as a two-piece form as described earlier. These species are hypothesized to have been established by a gene duplication event that formed a tandem repeat of the RNA genes, followed by the loss of the outer segment of each copy (Heinonen et al., 1987; Williams, 2002). Similarly, permuted tRNA genes might have originated from duplication of an intronic tRNA gene, followed by the loss of the outer exons to leave the 3′-half of the upstream tRNA gene and the 5′-half of the downstream tRNA gene (Figure 5A) (Soma et al., 2007; Di Giulio, 2008; Maruyama et al., 2010). In algae and archaea, these rearranged tRNA genes could have persisted in the genome because of the use of the upstream promoter-dependent transcription system and the tRNA maturation system that allows processing of permuted pre-tRNAs. In this context, the high frequency of permutation with the junction at position 37/38 (type II) can be ascribed to the overall dominance of introns located at the corresponding position in both eukaryotes and archaea. It is noteworthy that some tandem repeats of tRNA genes composed of single tRNA species containing an intron have been found in the nuclear genomes of green algae, namely the prasinophyte *O. lucimarinus* and the chlorophycea *Chlamydomonas reinhardtii*, which contain some and no permuted tRNAs, respectively, (Table 1) (Maruyama et al., 2010). Furthermore, an additional 5′-half is located downstream of the 5′-half of the permuted tRNA^Cys^(GCA) gene in the nuclear genome of *O. lucimarinus*. These duplicated tRNA genes may be structurally identical to the plausible intermediate stage of permuted tRNA evolution shown in the proposed model.

An alternative scenario is that the formation of permuted or circularized tRNA molecules preceded that of the corresponding permuted genes. Canonical pre-tRNAs co-transcribed from two tandemly-repeated intronic tRNA genes might be able to form a permuted pre-tRNA via the combination of the 3′-half of the initial tRNA and the 5′-half of the duplicated tRNA (Figure 5B). In support of this concept, a recent study showed that an artificial transcript simulating a tandemly-repeated intron-containing pre-tRNA could form a permuted tRNA structure *in vitro* (Tocchini-Valentini and Tocchini-Valentini, 2012). Furthermore, a circular pre-tRNA may be produced by ligation of an intron-containing (non-permuted) pre-tRNA at the acceptor-stem (Figure 5C). Indeed, many kinds of circularized non-coding RNAs have been identified in archaeal cells, indicating that the circularization of RNA is fairly prevalent, although the significance of this feature is still unknown (Danan et al., 2012). The resulting permuted or circular pre-tRNA molecules might have been reverse transcribed and integrated back into the genome to generate permuted tRNA genes (Figures 5B,C). Therefore, it is plausible that circular permutation has contributed to the evolution of the tRNA-like structures that are prevalent in nature (Pan et al., 1991; Pan and Uhlenbeck, 1993; Florentz and Giegé, 1995), and the cloverleaf structure of tRNA might have developed as a circularly permuted RNA isomer.

Regardless of the mechanism(s) by which permuted tRNA genes originated, the BHB motifs must have played a pivotal role during their development. The existence of a number of BHB-mediated *cis*-spliced tRNAs in algae and archaea may reflect a background that has accelerated the production of permuted tRNA genes. If so, permuted tRNA genes could have occurred frequently in archaea, especially in Crenarchaeota, whose splicing machinery can process various types of BHB motifs. However, only two permuted tRNA genes have been identified from one crenarchaeon (*T. pendens*) (Table 1), which harbors plenty of intron-containing tRNA genes (Fujishima et al., 2011). In eukaryotes, tRNA genes contain an intron at the canonical 37/38 position and tRNA genes of plants and yeast can be transcribed depending on the upstream promoter; therefore, it is plausible that eukaryotes could possess permuted tRNA genes with the junction at the canonical 37/38 position. However, most eukaryotes do not contain permuted tRNAs. These observations may indicate that the background for the development of permuted tRNA genes is intrinsically different among organisms. Moreover, even if permuted tRNA genes did once emerge in archaeal and eukaryotic species, they may not have been maintained in the genome due to their instability or harmful influence. For example, an inverted tRNA gene structure might have been lost easily, or the BHB motifs may have been associated with a specific adverse effect on the genome or organism.

The phylogenic distributions of BHB-mediated disruptions of tRNA genes are biased and an organism harboring all three types of disrupted tRNAs has not yet been identified. Some archaea, including *N. equitans* and *Caldiviga maquilingensis*, harbor split tRNAs but only a few *cis*-spliced tRNAs and no permuted tRNAs (Chan et al., 2011; Fujishima et al., 2011). Other archaea, including the Pyrobaculum and Thermofilum genera, harbor a number of intronic tRNAs that are disrupted at various positions, although Pyrobaculum have no split or permuted tRNAs (Fujishima et al., 2011). Similarly, green algae contain some permuted tRNAs but almost no ectopic intron-containing tRNAs (Kawach et al., 2005; Maruyama et al., 2010). Hence, *C. merolae* is unique because it possesses a number of permuted tRNAs and various intron-containing tRNAs. *C. merolae* might be permissive for the absorption and retention of various tRNA genes, or some characteristics of *C. merolae* may have accelerated the development and preservation of permuted tRNA genes during evolution. Considering that *C. merolae* has a compact genome, it is possible that the successive genome size reduction put pressure on redundantly duplicated tRNA genes to be arranged into a single permuted tRNA gene. Split tRNAs have not been identified in *C. merolae*, despite its potential ability to express them. Formation of a split tRNA may be a less efficient strategy to reduce the genome size because it requires two sets of promoter and terminator sequences to produce one species of tRNA. To date, there has been no report of an organism in which split tRNAs coexist with permuted tRNAs; therefore, the individual mechanisms and requisite elements required for the acquisition or maintenance of each disrupted tRNA gene could be substantially different, as suggested previously (Chan et al., 2011). This hypothesis supports a non-monophyletic origin of BHB-mediated disrupted tRNA genes, which may have arisen and disappeared multiple times independently in various organisms. The next challenge will be to identify the specific characteristics and fundamental background that led to the BHB-mediated disruption of tRNA genes, and to clarify the method of formation of each type of tRNA gene.

## Conclusions

The identification of circularly permuted tRNA genes has revealed a unique style of gene structure and RNA processing. Comparative genome analyses should be performed to identify more examples of permuted genes and to investigate the origin of the permuted tRNAs in correlation with other BHB-mediated disrupted tRNAs and mobile elements that target tRNA genes. Studies of the transcription and maturation systems for tRNAs that must have co-evolved with disrupted tRNA genes would help to clarify the physiological meaning and the mechanisms that govern the development and maintenance of permuted tRNA genes.

## Author contributions

Akiko Soma wrote the manuscript, and prepared Figures 1–5 and Table 1.

### Conflict of interest statement

The author declares that the research was conducted in the absence of any commercial or financial relationships that could be construed as a potential conflict of interest.
